# Persurf, a New Method to Improve Surfactant Delivery: A Study in Surfactant Depleted Rats

**DOI:** 10.1371/journal.pone.0047923

**Published:** 2012-10-17

**Authors:** Wolfram Burkhardt, Stephan Kraft, Matthias Ochs, Hans Proquitté, Lars Mense, Mario Rüdiger

**Affiliations:** 1 Department for Neonatology and Pediatric Intensive Care Medicine, Klinik für Kinderheilkunde, Universitätsklinikum Carl Gustav Carus, Medizinische Fakultät der Technischen Universität Dresden, Dresden, Germany; 2 Department for Pediatric Surgery, Klinikum Mutterhaus der Borromäerinnen, Trier, Germany; 3 Institute of Functional and Applied Anatomy, Hannover Medical School, Hannover, Germany; 4 Clinic for Neonatology, Charité, Universitätsmedizin Berlin, Campus Mitte, Berlin, Germany; University of Giessen Lung Center, Germany

## Abstract

**Purpose:**

Exogenous surfactant is not very effective in adults with ARDS, since surfactant does not reach atelectatic alveoli. Perfluorocarbons (PFC) can recruit atelectatic areas but do not replace impaired endogenous surfactant. A surfactant-PFC-mixture could combine benefits of both therapies. The aim of the proof-of-principal-study was to produce a PFC-in-surfactant emulsion (Persurf) and to test in surfactant depleted Wistar rats whether Persurf achieves I.) a more homogenous pulmonary distribution and II.) a more homogenous recruitment of alveoli when compared with surfactant or PFC alone.

**Methods:**

Three different PFC were mixed with surfactant and phospholipid concentration in the emulsion was measured. After surfactant depletion, animals either received 30 ml/kg of PF5080, 100 mg/kg of stained (green dye) Curosurf™ or 30 ml/kg of Persurf. Lungs were fixated after 1 hour of ventilation and alveolar aeration and surfactant distribution was estimated by a stereological approach.

**Results:**

Persurf contained 3 mg/ml phospholipids and was stable for more than 48 hours. Persurf-administration improved oxygenation. Histological evaluation revealed a more homogenous surfactant distribution and alveolar inflation when compared with surfactant treated animals.

**Conclusions:**

In surfactant depleted rats administration of PFC-in-surfactant emulsion leads to a more homogenous distribution and aeration of the lung than surfactant alone.

## Introduction

Disturbances of pulmonary surfactant lead to alveolar collapse with subsequent respiratory insufficiency and poor oxygenation [1]–[4]. Mechanical ventilation re-opens atelectatic areas however, does not treat the underlying disease, e.g. impairment of the endogenous surfactant system.

Administration of exogenous surfactant is the standard procedure in preterm infants with respiratory distress syndrome (RDS). Surfactant therapy has also been tested in other patients with respiratory insufficiency (such as acute respiratory distress syndrome – ARDS) [5], [6], however, results were less promising [7]. One possible explanation for the low efficacy of exogenous surfactant is the inhomogenous aeration in ARDS patients with a close proximity of atelectatic and overinflated areas and a probable inactivation of surfactant in alveoli due to inflammation. Since exogenous surfactant preferably distributes in open areas [8], [9], alveolar recruitment is required prior to surfactant therapy [10] to prevent a further increase in inhomogeneities.

Perfluorocarbons (PFC), that are used for liquid ventilation (LV), have a high density and can easily recruit atelectatic areas [11]. In PFC filled lungs end-expiratory collapse is prevented and a homogenous inflation of the lung is achieved. However, underlying disturbances of endogenous surfactant persist and respiratory insufficiency will reappear after evaporation of PFC.

Both interventions, LV and surfactant administration, were used concomitantly to re-open atelectatic areas and to substitute the disturbed surfactant. In animal studies surfactant was administered either after priming with PFC [12] or prior to LV [13]–[18], results, however, were controversial and less promising. The successive use of PFC and surfactant was not able to combine the potential benefits of both treatments and could not cause histomorphological or clinical improvement. Instead of using both interventions separately, a mixture of exogenous surfactant and PFC could combine advantages of both strategies and minimize side effects. The PFC component of the mixture recruits atelectatic areas without disturbing gas exchange and acts as carrier to facilitate the homogenous distribution of exogenous surfactant.

To test the former hypothesis in the present study, different emulsions of PFC and surfactant were produced. In a proof-of-principle-study the surfactant PFC mixture was given to surfactant depleted rats to compare short term effects (1 hour) with surfactant or PFC treatment. Primary outcome parameters were the histological evaluation of pulmonary surfactant distribution and aeration.

## Materials and Methods

### Emulsions of surfactant and PFC

Two different types of emulsion can be achieved, either PFC-in-surfactant or surfactant-in-PFC. To produce a PFC-in-surfactant emulsion (Persurf) diluted surfactant was mixed with PFC at a ratio (v/v) of 4∶1 [19]. Three different PFC were studied that have already been used previously [19], [20]: PF5080 obtained from 3M, Neuss, Germany; Rimar 101 (RM101) obtained from Miteni, Milano, Italy; and Perfluorooctylbromide (FO6167) obtained from ABCR, Karlsruhe, Germany. Curosurf™ was diluted in sterile saline to obtain different PL-concentrations, ranging between 1 and 20 mg/ml. After mixing Curosurf™ solution and PFC vigorously for 60 seconds, two distinct liquid phases can be separated. The supernatant – representing the remaining Curosurf™ solution – is removed. The remaining lower phase represents the PFC-in-surfactant emulsion. To determine the phospholipid (PL) concentration, lipids were extracted according to Bligh and Dyer [21] and the content of organic phosphorus was determined as described previously [20], [22]. To test the stability of Persurf, the PL-concentration was measured after 24 and 48 hours of storage at room temperature.

A surfactant-in-PFC emulsion was produced by adding undiluted Curosurf™ to PFC. The mixture was than exposed to ultrasonic energy (30 times, 0.6 sec impulse of 340 W). Different amounts of surfactant and three different PFC were tested. PL-concentration in emulsions was determined as described above. Fine structure of surfactant-in-PFC and PFC-in-surfactant emulsions was assessed by light microscopy after colouring surfactant with green dye [10], [23]. Stability during administration was tested in-vitro by giving 1.5 ml of green dye-coloured Persurf (20 mg/ml Surfactant concentration) over 60 seconds through a 8 Ch suction catheter.

### Ethics Statement

The study was approved by the local Review Board (Charité Berlin, Germany; no approval ID assigned). Care of the animals was in accordance with guidelines of ethical animal research. Rats were anesthetized with Ketamin and Pentobarbital intraperitoneally before beginning further preparation.

### Preparation of the animals

Male Wistar rats (age 2 month) were prepared as described previously [24]. A tube with side port was inserted via tracheostomy. Animals were placed on a pressure controlled ventilation (Bear Cube BP 2001 Infant Ventilator: Bear Medical Systems, Inc., Palm Springs, Calif., USA) with the following settings: positive inspiratory pressure (PIP) 12 cmH_2_O, positive end-expiratory pressure (PEEP) 3 cmH_2_O, fraction of inspired oxygen (FiO_2_) 1.0, inspiratory time 0.57 sec, respiratory rate 35/min.

Animals were surfactant depleted according to the protocol by Lachmann and coworkers [25]. Briefly, PIP and PEEP were raised to 26 and 6 cmH_2_O, respectively. 10 ml of warmed saline (approximately 30 ml/kg body weight) were instilled into the lung and suctioned outthereafter. The procedure was repeated at least 5 times and an arterial blood gas was obtained. If the PaO_2_ was above 100 mmHg, the procedure was repeated. If values below 100 mmHg were achieved, lavage procedure was stopped. To exclude a spontaneous improvement, blood gases were repeated after 30 min, if PaO_2_ remained below 100 mmHg, the experimental protocol was started. The efficacy of that method to remove alveolar surfactant has been studied recently [24].

### Experimental protocol

**Figure 1:**
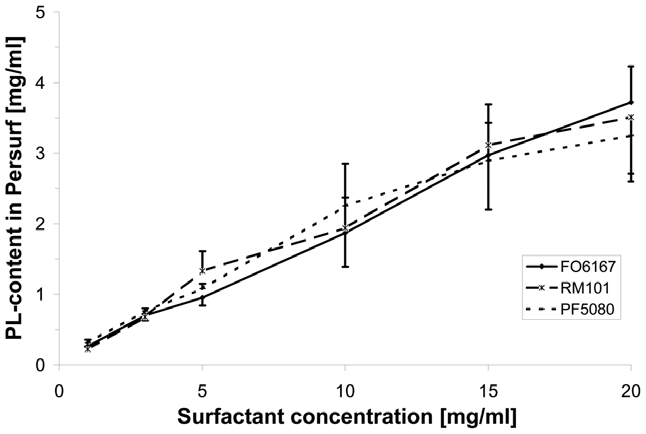
Phospholipid concentration in PFC-in-surfactant emulsion (Persurf). Shown are the mean ± standard deviation of phospholipid (PL) content in Persurf produced with different surfactant concentrations and with three different PFC.

Surfactant was stained as described by Krause and coworker [10], [23]. In short, a diluted (1∶10) green histological dye (Green Tissue Marking Dye, WAK-Chemie Medical, Bad Soden, Germany) was mixed with the surfactant preparation at a ration of 1∶10 (v/v). Since PF5080 has been used in previous animal experiments [26], [27], PF5080 was used with the stained surfactant to prepare Persurf at a final PL-concentration of 3.3 mg/ml (fig. 1).

Animals were randomized into three groups: *Surfactant* (n = 10) animals received 100 mg/kg of stained Curosurf™ as a bolus; *Persurf* (n = 10) animals received the Persurf mixture at an amount of 30 ml/kg at a rate of 1.5 ml/min; *PFC* (n = 10) animals received 30 ml/kg of PF5080 at the same rate. Each mixture was administered via the side port. Ventilation of the animals was continued without changing ventilatory settings.

Samples (150 µl) of arterialized blood were drawn to determine blood gases (ABL 505, Radiometer Med. A/S, Denmark) both, prior to and after lavage, and 5, 10, 20, 30, 60 minutes after intervention.

Animals were sacrificed by an overdose of potassium chloride and pentobarbital at the end of the experiment at 1 h. Rat lungs were prepared and fixed in situ at the time of sacrifice, while a constant PEEP of 5 cmH_2_O was maintained. A sternotomy was made to expose lungs and heart. The left atrium was incised to allow sufficient drainage of the perfusate. Phosphate buffered saline was injected into the right ventricle to flush the pulmonary circulation free of blood. For perfusion fixation of the lung, 4% paraformaldehyde was instilled via the right ventricle with a constant pressure of 20 cmH_2_O for 20 minutes. The lung was removed and submersed in fixative for 24 hours [28].

### Histological evaluation of surfactant distribution

Lung tissues were embedded in paraffin in a way that allowed to distinguish between right and left lung and between upper, median and lower part of each lung. Sections of 4 µm thickness were stained with hematoxylin and eosin. From each animal, sections from 5 tissue blocks obtained by systematic uniform random sampling were examined in a blinded manner by light microscopy [24], and point and intersection counting were performed according to established stereological methods [29], [30] to estimate the volume fraction of gas-filled (Surfactant group) or PFC-filled areas (PFC/Persurf group) within lung parenchyma and the surface fraction of alveolar epithelium covered with green dye, respectively. The mean number of points or intersections counted per lung was 400 to ensure that the total observed variability within a group is largely dominated by the biological variability between individuals and not by the variability due to stereological sampling [31].

### Statistics

Data were analysed using SPSS software. Analysis of variance (ANOVA) for repeated measures was used to assess differences among groups in change over time. A p<0.05 was considered as statistically significant.

## Results

### Emulsions of surfactant and PFC

As shown in figure 1, the concentration of PFC-in-surfactant emulsion (Persurf) depends on the surfactant-concentration of the Curosurf™ solution. A final concentration between 3 and 4 mg/ml of PL-content in Persurf can be achieved using Curosurf™ at a concentration of 20 mg/ml. The achieved PL-concentrations did not differ between the three different PFC. As shown in table 1, the PL-concentration remained stable of slightly increased during the studied period of 24 and 48 hours.

**Table 1:** Stability of PFC-in-surfactant emulsions (Persurf).

Surfactant-concentration [mg/ml]	Type of PFC	PL-concentration in Persurf [mg/ml]		
		*0 hours*	*24 hours*	*48 hours*
**1 mg/ml**	*FO6167*	0.26±0.01	0.29±0.07	0.43±0.03*
	*RM101*	0.22±0.03	0.28±0.02	0.36±0.13
	*PF5080*	0.32±0.05	0.43±0.18	0.36±0.04
**3 mg/ml**	*FO6167*	0.7±0.07	0.72±0.3	0.92±0.19
	*RM101*	0.68±0.04	0.82±0.16	1.08±0.36*
	*PF5080*	0.76±0.04	0.83±0.02	1.39±0.51*

Shown are the mean ± standard deviation of phospholipid (PL) concentrations in Persurf produced from two different surfactant concentrations (1 vs. 3 mg/ml) after 0, 24 and 48 hours in room air.

* P<0.01 vs. 0 hours.

PL-concentrations in a Surfactant-in-PFC emulsion were significantly lower than in PFC-in-Surfactant emulsions and did not correlate with the initial surfactant concentration (table 2). Highest PL-values were achieved with FO6167. The emulsions were not stable, thus almost no PL was measured after 24 and 48 hours (data not shown).

**Table 2:** Surfactant-in-PFC emulsions.

Amount of surfactant [mg]	PL-concentration in Surfactant-in-PFC emulsion [mg/ml]		
	*FO6167*	*RM101*	*PF5080*
**4**	1.3±0.01	0.48±0.08	0.32±0.02
**12**	1.02±0.03	0.42±0.02	0.16±0.002
**20**	1.57±0.5	0.13±0.004	0.24±0.01

Shown are the mean ± standard deviation of phospholipid (PL) concentrations in Surfactant-in-PFC emulsions produced from three different surfactant quantity (4, 12, 20 mg) and three different PFC. The PL-concentrations are significantly (P<0.01) higher in FO6167 containing emulsions than in the other two.

**Figure 2:**
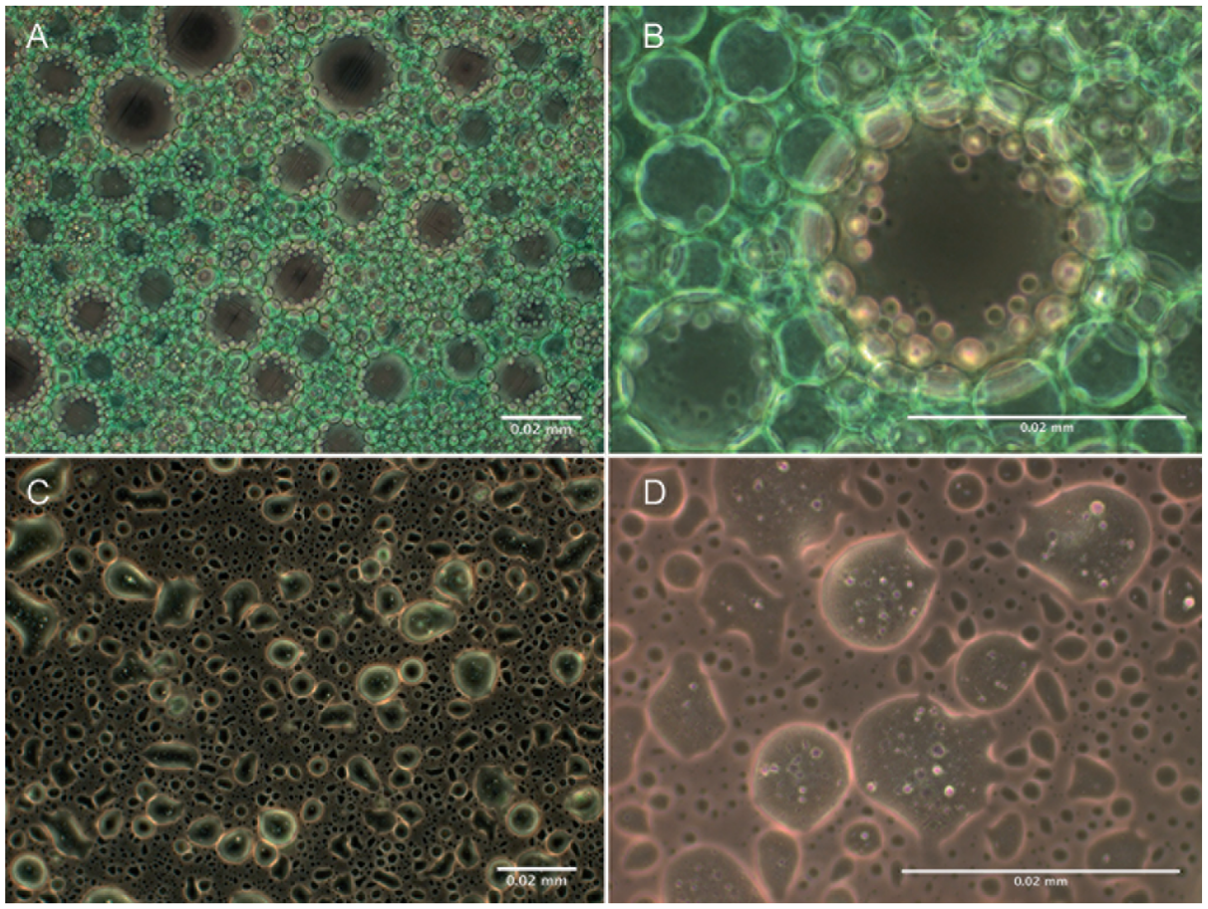
Ultrastructure of PFC-in-surfactant and Surfactant-in-PFC emulsions. Shown are PFC-in-surfactant emulsion (20 mg/ml Surfactant) at low (A) and high (B) magnification and Surfactant-in-PFC emulsion (20 mg Surfactant; C, D). Surfactant is coloured using green dye.

**Figure 3:**
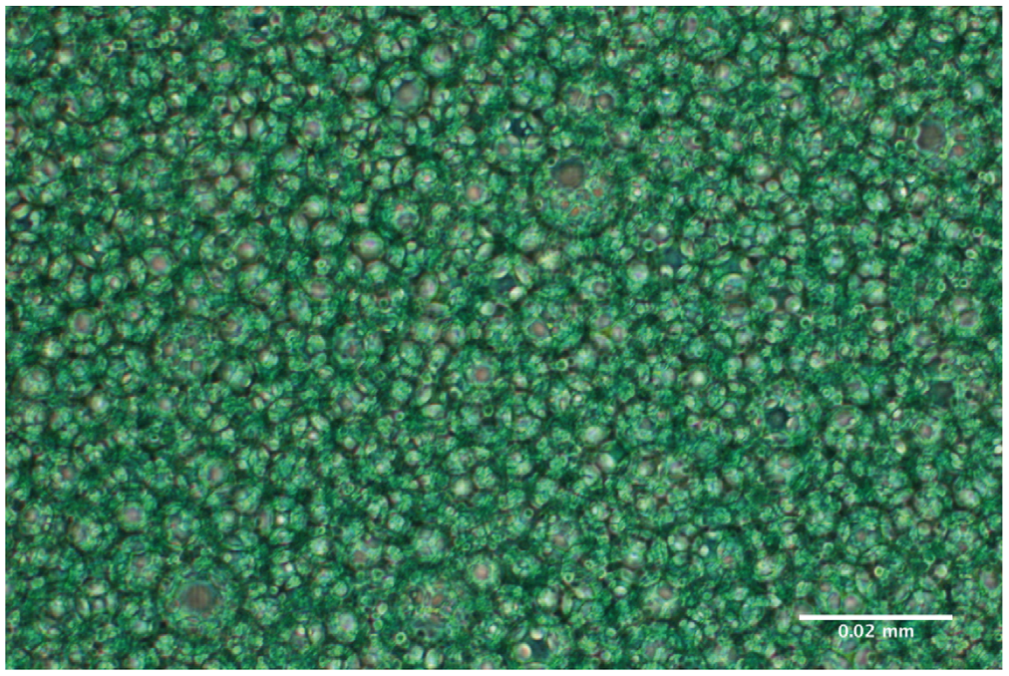
Ultrastructure of PFC-in-surfactant emulsion after administration through suction catheter and collection in a vial. Surfactant was coloured using green dye.

Emulsions of green dye coloured surfactant and PFC revealed the differing fine structure. Droplets containing dispersed PFC (PFC-in-surfactant emulsion) or Surfactant (Surfactant-in-PFC emulsion) were visible (figure 2). After administering Persurf through a suction catheter and collecting the fluid in a vial, by macroscopic view no phase separation could be determined. Light microscopy revealed unaltered fine structure after administration (figure 3). Samples from lower and upper parts of the vial showed only low variance.

### Animal data

**Figure 4:**
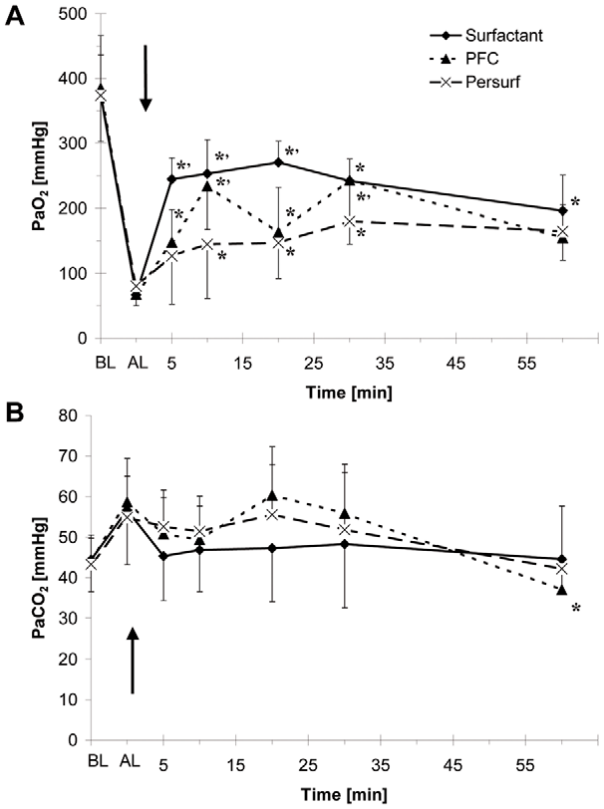
Blood gases. Shown are the mean ± standard deviation of (**A**) PaO_2_ and (**B**) PaCO_2_ of animals treated with *PFC*, *Surfactant* or *Persurf* before (BL) and after lavage (AL) and at different time points after therapy (arrow). * P<0.01 vs. after lavage; # P<0.01 vs. Persurf.

All animals survived the experiment. There were no significant differences in vital parameters during the experimental period or between the groups (data not shown). The effect of treatment on oxygenation differed between the three groups. Whereas the anticipated immediate improvement of PaO_2_ was found after *Surfactant* administration, the improvement in oxygenation was more slowly in *PFC* and *Persurf* treated animals. At the end of the experiment there were no differences in PaO_2_ between the three groups (figure 4A). PaCO_2_ did slightly increase after lavage. Therapeutic interventions were associated with only minor, non significant changes in PaCO_2_. There were no differences between groups (figure 4B).

### Histological evaluation of aeration and surfactant distribution

**Figure 5:**
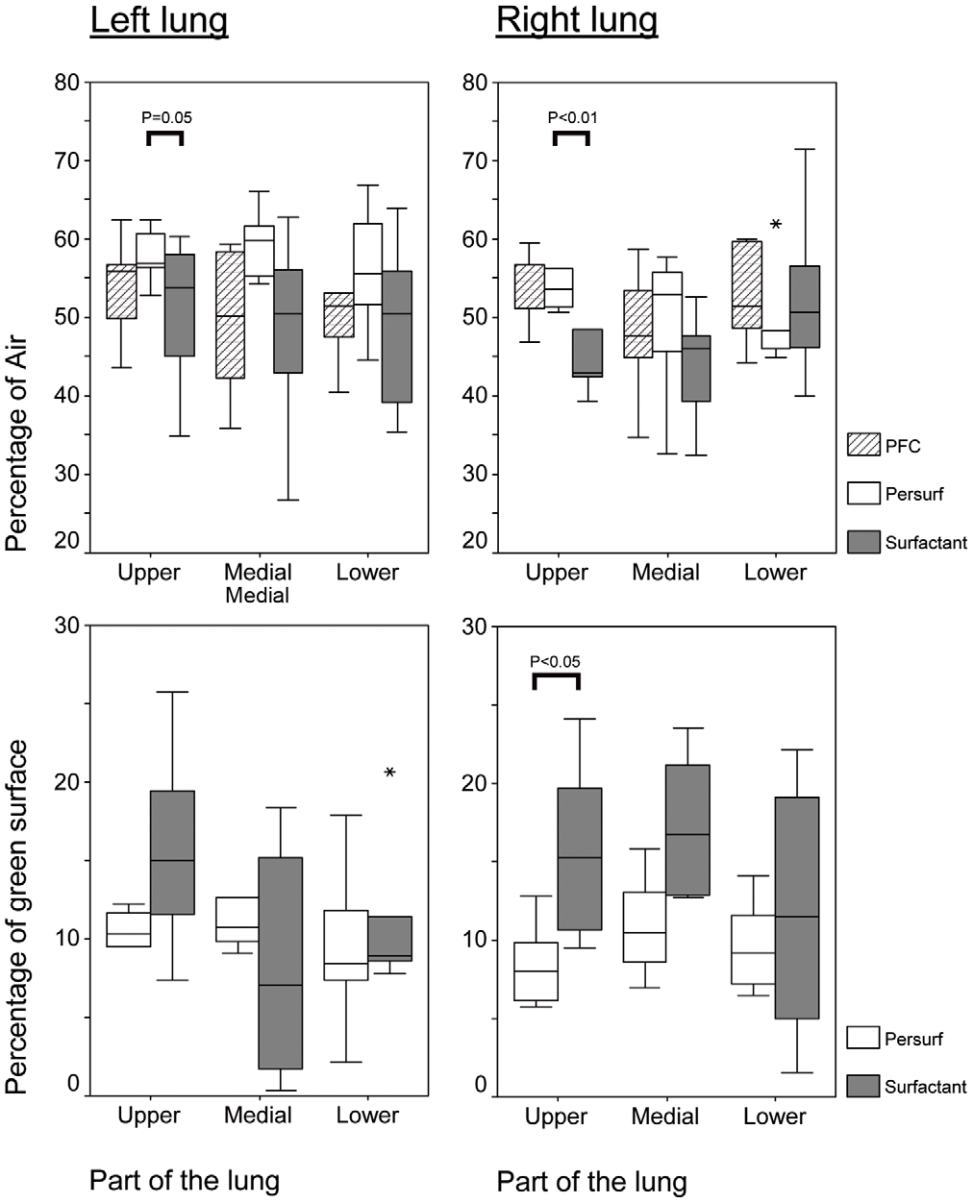
Histological parameters. The upper panel shows the percentage of gas filled areas (in Surfactant group) or fluid filled areas (in PFC/Persurf groups) on total parenchyma. The lower panel shows the percentage of green dye covered surface on total alveolar surface is shown. Values are shown as median, range and outlyers (*) for the upper, middle and lower part of the left and right lung.

When compared with *Persurf-*group (54.3±7%) overall pulmonary aeration was significantly (P<0.005) lower in *Surfactant*- (48±9%) but not *PFC*-animals (51±7%). Whereas gas distribution was similar in all parts of the lungs in *PFC-* and *Persurf*-animals (coefficient of variation 54 and 55%, respectively), larger variations (CV 99%) were found in *Surfactant*-animals (figure 5, upper part).

With respect to surfactant distribution, the total surfactant covered surface was significantly (P<0.05) larger in *Surfactant-* (13.2±7%) than in *Persurf*-animals (10.3±3%). However, large variations between and within different parts of the lungs were found in *Surfactant*-group (CV 53.3%). The variations (CV 11.7%) were less prominent in *Persurf*-animals (figure 5, lower part).

## Discussion

Whereas administration of exogenous surfactant is effective to recruit atelectatic alveoli in preterm infants with RDS, it is less effective in patients with ARDS since surfactant does not reach atelectatic areas [5], [7], [32], [33]. Partial liquid ventilation with PFC can open atelectatic areas, however, does not cure the underlying disease, e.g. disturbances of the pulmonary surfactant system. The advantages of both substances were used in the present study by producing an emulsion of PFC and surfactant. In the present proof-of-principle-study it was shown, that a PFC-in-surfactant emulsion (Persurf) (i) can contain a sufficient amount of surfactant, (ii) leads to a similar improvement of oxygenation like surfactant or PFC alone, and (iii) leads to a more homogenous distribution of surfactant in surfactant depleted rats.

### PFC-surfactant emulsions

In the past, therapy with PFC and surfactant were combined by either giving surfactant after PFC priming [12] or prior to liquid ventilation [14]–[18]. No data concerning a PFC-surfactant mixture have been published, because PFC are insoluble and immiscible and surfactant cannot be solved in PFC. Emulsions are the only chance to combine both, the lipophilic PFC and the amphiphilic surfactant. According to data of the present study, surfactant-in-PFC emulsions are less promising since the amount of surfactant was too low and the emulsion was not stable. In preliminary studies other techniques were tested to create stable surfactant-in-PFC emulsion, but were even less promising. The PFC-in-surfactant emulsion, however, did contain a substantial amount of surfactant. Considering a PFC filling volume of 30 ml/kg body weight and about 100 mg/kg of surfactant, the concentration of about 3 mg phospholipids per ml of PFC would be appropriate. During conventional surfactant administration most of the surfactant does not reach the alveolar level [9], and therefore it has been speculated that the actual alveolar requirement could be even as less as 4 mg/kg [34] if a sufficient surfactant distribution is achieved. In the present animal experiments the same total amount of Curosurf™ was used in the Persurf and Surfactant groups.

Whilst partial liquid ventilation can be performed with less than 30 ml/kg PFC volume, PFC serves as a carrier solution for surfactant in Persurf. For a homogenous surfactant distributation a complete filling of the functional residual capacity with 30 ml/kg Persurf is necessitative.

In vivo studies suggest that surfactant's structure and function are not impaired by PFC [35], [36]. In vitro studies show that PFC lower the spontaneous endogenous surfactant exocytosis in type II alveolar cells but not the induced one which does not seem to be of clinical importance [37].

Regarding the fine structure of PFC-in-surfactant and Surfactant-in-PFC emulsions we conclude that phospholipids serve as dispersants at the interface of PFC and surfactant. Phospholipids will be orientated with their lipophilic faty acids towards PFC and their hydrophilic parts towards the aqueous phase.

Beside lipid concentration, stability of the emulsion represents an important issue. On one hand, the mixture should have a certain stability for storage and intratracheal administration. On the other hand, the emulsion has to disintegrate in the lung to allow the undisturbed evaporation of PFC. In the present study Persurf disintegrated upon shaking into surfactant and PFC; if it was not shaken it remained stable for at least two days. Significantly higher phospholipid concentrations in three samples after 48 hours might be a consequence of PFC-evaporation and do not limit applicability. Sufficient stability of Persurf during administration could be proven by in-vitro experiments and light microscopy. Thus, the properties of Persurf seem to meet the clinical requirements although bed-side preparation of Persurf seems to be necessary. In consequence, no stabilizer – as it has been described for PFC containing blood substitutes (PFC-in-phospholipid emulsions) – was tested in the present study.

### Persurf administration does not deteriorate gas exchange

Both, PFC or surfactant improved oxygenation, an effect that has been described previously in the used lavage model [17], [25]. In the present study, the oxygenation was also improved by Persurf administration. The Persurf effect was not as fast as for surfactant, but comparable to the PFC effect. The difference is explained by differences in application time. Whereas surfactant was given as a bolus, the complete administration of PFC and Persurf required about 8 minutes. Since bolus administration of surfactant or PFC are associated with deterioration in cerebral oxygenation [26], [38], [39], the slow improvement in blood gases is preferable.

Ventilation of the animals was standardized in this study. A low initial PaO2 is due to a strategy which was not optimized concerning oxygenation. Increasing the inspiratory peak pressure or ventilating in a volume controlled mode would result in a higher initial PaO2.

### Persurf improves surfactant distribution

Histological evaluation revealed a large variation in aeration in surfactant treated lungs, as it has been described before for animals with experimental ARDS [10]. In PFC and Persurf ventilated animals the lungs contained fluids instead of air at paraffin embedding. Thus, in contrast to surfactant treated lungs, the PFC filled lungs were more homogenous inflated, an effect that has been described previously [18], [40]. A similar improvement in alveolar expansion was found after Persurf treatment. Thus, PFC associated recruitment of atelectatic alveoli [11], was also achieved in Persurf treated animals. In liquid ventilated animals atelectasis will re-appear after evaporation of PFC, since the underlying disturbances of pulmonary surfactant persist [41]. In contrast, the homogenous alveolar expansion should persist in Persurf treated animals after PFC evaporation since surfactant has been replaced. However, further studies are required to prove that assumption. The present study was only designed to test short time effects and surfactant distribution of Persurf treated animals.

Histological evaluation of surfactant distribution showed more surfactant in the alveoli after surfactant than after Persurf administration. However, the distribution in surfactant treated animals was rather inhomogenous. A much more homogenous distribution was achieved by Persurf treatment which highlights the benefit of simultaneous PFC and surfactant administration. Similar effects could not be seen in surfactant treatment after PFC priming [12]. Concerning the lower amount of stained alveoli in Persurf treated animals two different explanations are possible. One could be an artificial effect associated with a wash out of Persurf and thus surfactant during the fixation process. Another explanation is that much of the surfactant remained in the large airways since PFC had not evaporated yet. Thus, it could be speculated, that after complete evaporation the transfer to the lower bronchi is increased and more surfactant is found in the alveoli. It should be emphasized that this effect cannot be seen due to the shortness of Persurf ventilation and was not an objective of this study.

### Limitations and clinical implication of the study

Further animal studies are required to understand Persurf administration and its limitations in more detail. The present study was performed as proof-of-principal and therefore, a simple animal model of surfactant depletion was used with some limitations. Firstly, Persurf administration was only tested in a rat model and not a large animal model. A rat model was chosen because surfactant can easily be given as a bolus and it was the aim to compare distribution of surfactant and Persurf. Due to the small size of the rats distribution of surfactant might be less of a problem than in larger animals. Secondly, in the present study only the short time effect on oxygenation and surfactant distribution was studied. Further studies are warranted, to investigate whether surfactant effect persists after evaporation of PFC. Thirdly, the lavage model was used to produce surfactant deficiency. Whereas the model is well established, it does not necessarily represent an ARDS model in its full complexity of pathophysiological factors [1]. Future studies in larger animals, however, will use a different model that mimics clinical situation in ARDS patients. The appropriate dosage of surfactant in ARDS patients has been long discussed. Assuming an application to the alveoli, as it is performed by our method, a dosage of 100 mg/kg may be sufficient. Due to surfactant inactivation a higher concentration could be necessary. There is a chance to administer more Persurf in accordance to the evaporation rate of PFC, or to increase the surfactant concentration in Persurf. Finally, a new surfactant preparation which is more resistant against inactivation could be used.

### Conclusion

The present study describes for the first time that a mixture of PFC and surfactant can be administered to treat surfactant deficiency in an animal model. The study offers new possibilities for research in improving lung function of ARDS patients. Using Persurf, the lung can be easily filled without any bronchoscopic administration [6] or discontinuation of the mechanical ventilation. Atelectatic areas are recruited and surfactant is delivered. After evaporation of PFC the exogenous surfactant might remain in the lung and prevents end-expiratory collapse. Thus, Persurf offers a good approach to consider surfactant therapy in pediatric and adult intensive care medicine.
